# Treatment of Complicated Henoch-Schönlein Purpura with Mycophenolate Mofetil: A Retrospective Case Series Report

**DOI:** 10.1155/2010/254316

**Published:** 2010-06-15

**Authors:** A. A. Nikibakhsh, H. Mahmoodzadeh, M. Karamyyar, S. Hejazi, M. Noroozi, A. A. Macooie, A. Gholizadeh, L. Gholizadeh

**Affiliations:** Department of Pediatric Nephrology, Urmia University School of Medicine, Iran

## Abstract

*Background*. Henoch-Schönlein purpura (HSP) is the most common childhood vasculitis with an incidence of approximately 10 per 100 000 children. There is some evidence to support steroid therapy in the treatment of severe abdominal pain, severe nephritis, and central nervous system involvement. However, the routine use of corticosteroids is controversial. Frequent relapses, lack of response to steroid, steroid dependency, and steroid side effects may occur in some patients. Mycophenolate mofetil (MMF) gains increasing popularity in the treatment of autoimmune disorders, but hitherto, the available evidence to support the use of MMF in HSP is limited to some case study reports. *Case Presentation*. We report six children with HSP who failed to respond to systemic steroid therapy, whereas MMF successfully treated the manifestations of the disease. *Conclusion*. The manifestations of HSP disappeared mainly during the first week of treatment with MMF and all the patients were in a complete remission at the end and after discontinuation of the therapy. In our experience, MMF appeared to be safe and effective for the maintenance of remission in the HSP patients.

## 1. Introduction

Henoch-Schönlein purpura (HSP) is the most common vasculitis in children with an incidence of approximately 10 per 100,000 children per year and is slightly common among boys (60%) than girls (40%) [1]. The diagnosis is based on the palpable purpura in the presence of one of either diffuse abdominal pain, a biopsy showing predominant immunoglobulin A (IgA) deposition, arthritis, or arthralgia and/or renal involvement (hematuria and/or proteinuria) [2]. Typically, patient is a young child presenting with clinical manifestations including palpable purpura, arthritis, abdominal pain and gastrointestinal bleeding. The etiology of HSP is mainly unknown and antecedent upper respiratory tract infection, usually viral, has been reported and Streptococcal infection has been emphasized as an important triggering factor [3]. Also it is known that IgA plays an important role in the pathogenesis of the disease [1]. Most cases are self-limiting and require no treatment apart from symptomatic relief, but recurrence of symptoms occurs in about 33% of the cases. It seems that relapse often occurs between two weeks and 18 months after initial resolution of symptoms and children with kidney involvement are more likely to have recurrences [4]. In some patients, nephritis occurs due to IgA deposition in the renal mesangium [1]. More serious complications such as the involvement of central nervous system, renal failure, and intussusception may also occur. There is no consensus on the optimal treatment in the case of significant renal or other organs involvement where treatment may have a significant impact on the long-term outcome. 

The routine use of corticosteroids in HSP is controversial. In a randomized placebo controlled study of oral prednisolone, abdominal symptoms were more prolonged in the prednisolone group. Further, there was no significant difference in the development of gastrointestinal complications the exception was the development of two intussusceptions in the placebo group and none in the steroid group [5]. On the contrary, some retrospective studies and case reports have suggested a benefit of steroids such as prednisolone in the treatment of abdominal pain [6, 7], HSP nephritis, and as prophylaxis for nephropathy [8, 9]. Then, the treatment of HSP nephritis remains controversial [4]. HSP nephritis accounts for 1.8–3% of children with chronic kidney disease [10] and the presence of nephrotic/nephritic syndrome has been associated with a poor prognosis [4]. Long-term renal impairment occurs in 19.5% of those with a history of nephritic or nephrotic syndrome [11] which may imply a necessity for a more aggressive treatment to improve the outcome. 

Mycophenolate mofetil (MMF), an immunosuppressive agent, has been widely used in the organ transplantation including pediatric renal transplantation [12]. MMF also appears to be a promising therapeutic agent in many autoimmune diseases such as lupus nephritis and in IgA nephropathy which is a form of primary glomerulonephritis [13]. Hitherto, the available evidence to support the use of MMF in HSP is limited to some case study reports [14]. 

In this case series study, we report the benefit effect of MMF in the treatment of some complicated HSP cases. The study subjects include six patients admitted into pediatric nephrology ward in a public hospital in Urmia-Iran. The patients were admitted with the diagnosis of HSP from March 2007 through March 2008. All the patients had been administrated steroid initially to control their complications including severe abdominal pain, gastrointestinal bleeding, and severe nephritis. Subsequently, medication changed to MMF regarding the following criteria (1) unresponsive to steroid, (2) steroid side effects, and (3) steroid dependency (two times of relapse during steroid tapering).

## 2. Case Series

### 2.1. Case Number 1

A 6-year-old boy referred to our hospital with the exacerbation of HSP symptoms including typical diffuse palpable purpuric and hemorrhagic lesions, abdominal pain, and arthritis. The patient had moon face, body weight gain, relapse of gastrointestinal bleeding, and severe abdominal pain despite taking prednisolone for eight weeks (2 mg/kg/day for the first four weeks and 2 mg/kg/every other day for the last four weeks). Abdominal sonography was normal. Antistreptolysin-O (ASO) titer was 250 Todd, C reactive protein (CRP) was qualitatively positive (+2), erythrocyte sedimentation rate (ESR) was 55 mm/h, and Antineutrophil cytoplasmic antibody (ANCA) as well as antinuclear antibodies (ANAs) yielded negative results. We tapered off the prednisolone gradually and switched to MMF 30 mg/kg/day. The gastrointestinal bleeding stopped during the first 48 hours of treatment and all symptoms and cutaneous manifestations resolved during the first week. The treatment continued for the next three weeks. There was no evidence of relapse in a 6-month follow-up.

### 2.2. Case Number 2

A 21-month-old boy presented with typical diffuse palpable purpuric and hemorrhagic lesions, hematuria, gastrointestinal bleeding, abdominal pain, and arthralgia. Abdominal CT scan and sonography were normal. ASO titer was 166 Todd, CRP was +1, and ESR was 61 mm/h. Despite treatment with prednisolone for two months (2 mg/kg/day for the first month and 2 mg/kg/every other day for the second month), there was no lasting improvement in HSP symptoms (abdominal pain, arthralgia, purpuric and hemorrhagic lesions). Gastrointestinal bleeding was exacerbated and two episodes of relapse observed. He had moon face and body weight gain. Steroid therapy changed to MMF 30 mg/kg/day. Abdominal pain and gastrointestinal bleeding resolved in one week. Treatment with MMF was continued for the next two weeks and then tapered off in the next month. There was no evidence of relapse in a 6-month follow-up.

### 2.3. Case Number 3

A 2-year-old boy presented with typical diffuse palpable purpuric and hemorrhagic lesions, ankle and elbow arthritis, hematuria, gastrointestinal bleeding, and abdominal pain. Abnormal laboratory tests were CRP = + 3, ESR = 42 mm/h. Abdominal sonography and other laboratory tests were normal. The patient was under steroid therapy for eight weeks (2 mg/kg/day) and he had moon face and body weight gain. With tapering steroid, exacerbation and recurrence occurred for three times (steroid dependency). MMF 30 mg/kg/day was administered and tapering of steroid commenced and discontinued after three weeks. All the clinical manifestations resolved in one month of the treatment and MMF was tapered during the next month. There was no evidence of relapse over one-year follow-up.

### 2.4. Case Number 4

A 6-year-old boy was referred to our hospital with HSP symptoms exacerbation. The patient had palpable purpura, gastrointestinal bleeding, abdominal pain, and arthralgia in spite of taking prednisolone (2 mg/kg/day) for weeks. Abnormal laboratory tests were ASO = 333 Todd, CRP = +1, ESR = 29 mm/h. Abdominal sonography and other laboratory tests were normal. We administered MMF 30 mg/kg/day while tapering steroid therapy gradually. Treatment with MMF continued for one month and tapered off within the next month. There was no evidence of recurrence over one-year follow-up.

### 2.5. Case Number 5

A 7-year-old girl referred to our hospital due to developing nephrotic level of proteinuria (above 2 gr/day) and microscopic hematuria following steroid tapering. Before admission to our hospital, the patient had diffuse palpable purpuric and hemorrhagic lesions, arthritis, generalize edema, microscopic hematuria, gastrointestinal bleeding, and severe abdominal pain. At the beginning of the disease, Methylprednisolone pulse (20 mg/kg/day) had been started intravenously for three times (every other day) and continued with oral prednisolone 2 mg/kg/day for the first month and 2 mg/kg/every other day for the second month. 

Abnormal laboratory findings were CRP = +3, ESR = 74 mm/h; see Table 2. Other laboratory tests including serum lipids, serum albumin, ASO, ANCA, ANA, C3, C4, BUN, and Scr were normal. Due to continuing significant proteinuria (above 2 gr/day) and microscopic hematuria, renal biopsy was performed and showed diffuse mesangial cell proliferation (light microscope) and predominant deposition of IgA in mesangial matrix and basement membrane of glomerulus (immunofluorescence microscope). After admission of the patient to our hospital, treatment with MMF 30 mg/kg/day commenced and the oral prednisolone (2 mg/kg/every other day) which had been started since 2 months ago was discontinued. 

After treatment with MMF for two months, proteinuria diminished from 2 gr/day to 130 mg/day, but microscopic hematuria continued. After six months, MMF was discontinued and after a six-month follow-up there was not any clinical and laboratory abnormality except mild microscopic hematuria.

### 2.6. Case Number 6

An 18-month-old boy referred to us due to steroid dependency and frequent recurrences while taping steroid. The patient presented with diffuse purpuric lesions, gastrointestinal bleeding, severe abdominal pain, and microscopic hematuria in spite of taking prednisolone (2 mg/kg/day for four weeks and 2 mg/kg/every other day for six weeks). The patient was readmitted again to hospital with gastrointestinal bleeding and arthritis for fourth times. Abdominal sonography and laboratory tests including ESR, CRP, and ASO were normal. After admission of the patient to our hospital, MMF 30 mg/kg/day commenced and steroid was tapered off gradually. All clinical manifestations resolved within one week and hematuria improved after one month of the treatment with MMF. Treatment with MMF was tapered off during the next month. There was no evidence of recurrence over one-year follow-up.

## 3. Discussion

MMF gains increasing popularity in the treatment of autoimmune disorders such as lupus nephritis, vasculitis, necrotizing glomerulonephritis, corticosteroid resistant glomerulonephritis, and IgA nephropathy [12, 13, 15]. It has been shown that MMF suppresses lymphocyte proliferation and decreases antibody production in the kidney diseases [13]. Recent studies suggest that mycophenolatic acid (MPA), the active metabolite of MMF, can significantly inhibit the adhesion of leukocytes to endothelial cells, which is a key process in the development of ANCA-associated vasculitis [16]. Hu et al. found that MMF was effective in controlling disease activity and improving renal function in Chinese patients with MPO-ANCA-associated vasculitis [16]. In another report, a patient with adult crescentic progressive glomerulonephritis in whom long-term complete remission was achieved after MMF therapy was discussed by Dede [13]. A case of HSP that manifested after pantoprazole ingestion has been reported recently by Muzaffar [17]. In this patient, the clinical manifestations including terminal ileitis and rapidly progressive glomerulonephritis were initially unresponsive to intravenous pulse steroids, whereas, his renal function recovered after treatment with MMF [17]. In a study, the clinical courses of six patients (four boys and two girls with mean age of 13.2 years) with HSP and nephrotic syndrome were reviewed [18]. In this study, the treatment protocols included oral prednisolone, and in the nonresponders included cyclosporine A, cyclophosphamide, MMF, or tacrolimus; see Table 3. They suggested that an early aggressive immunosuppressive approach improves long-term renal outcome in HSP patients with nephrotic syndrome [18].

Filler et al. described the pharmacokinetics of MMF in 15 pediatric patients with vasculitis and connective tissue diseases involving the kidney. Therapy was effective in inducing remission in four of nine patients with active diseases. Relapse occurred in only one of five other patients. All of six patients with controlled disease maintained remission. There were few side effects including diarrhea and leukocytopenia in one case and viral infections in two cases. They conclude that MMF at 900 mg/m^2^ per day appears to be effective in these patients [19].

In our case series study, we report six cases with HSP in whom steroid therapy failed to treat complications and keep in persistent remission so, medication switched to MMF (Table 1). All patients were closely followed up at least for six months for any complaint, symptoms, and signs of HSP such as palpable purpura, and arthritis. Blood pressure, serum creatinine level, complete blood count, platelet count, hematocrit, urine analysis, urine protein measurement, and side effects of MMF have been checked in the patients. In our study, MMF was found to be a useful immunosuppressant due to its fewer undesirable effects and good treatment effects for control of complication and relapse of HSP. 

There was not any significant side effect, except mild anemia in the case 2 (not required any intervention) and mild diarrhea in the case 3 which relived by changing MFM to four divided doses. HSP manifestations disappeared mainly during the first week of treatment with MMF. With the exception of one patient with persistent hematuria for a long time, all the patients achieved complete remission at the end and after discontinuation of the therapy. 

In our experience, MMF appeared to be effective for maintenance of remission in HSP patients. 

There is some evidence to support steroid therapy in the treatment of severe abdominal pain, gastrointestinal bleeding, severe nephritis, and central nervous system involvement [20]. However, as seen in our patients, frequent relapses, lack of response to steroid, steroid dependency, and steroid side effects have been reported [14, 15]. In literature the other treatment choices in complicated and sever HSP patients varied from combined heavy immunosuppressant (Cyclosporine, Cyclophosphmide) therapy to plasmapheresis [21]. So, we considered a more specific alternative to steroid therapy for HSP patients. 

On the other hand, considering favorable experiences with MMF such as less adverse effects than other immunosuppressive drugs, benefit effects in the treatment of many immunologically mediated renal diseases, and the antifibrotic and antiproliferative effects of it [22, 23], it is suggested that MMF can be especially valuable in the treatment of the complicated HSP. 

According to this case series study, MMF can be a safe and effective medication in the treatment of the complicated HSP. So, therapy could be changed to MMF considering the following criteria: unresponsive to steroid, steroid side effects, and steroid dependency (two relapses during steroid tapering). However, there are some important factors that may influence the results of our study including the possibility of spontaneous improvement of HSP without intervention, therapeutic effects of antecedent steroid before starting MMF. It is important to point out that we do not claim MMF works in all patients and some patients with HSP may fail MMF and may require more aggressive treatment. 

So, more controlled prospective studies are necessary to prove the efficacy of this medication in the HSP treatment.

## 4. Conclusion

The results of this case series study suggest that mycophenolate mofetil would be a promising therapeutic alternative in the treatment of the complicated HSP. However, multicentre clinical trials with long-term followup will be necessary to confirm effectiveness of MMF in the treatment of the complicated HSP patients.

## Figures and Tables

**Table 1:** Demographic features of patients.

	AGE	Sex	Presenting signs and symptoms
Case 1	6 yr	Male	Palpable purpura, Hemorrhagic purpura, Arthritis, Abdominal pain

Case 2	21 month	Male	Palpable purpura, Hemorrhagic purpura, Arthralgia, Abdominal pain, Hematuria, Gastrointestinal bleeding

Case 3	2 yr	Male	Palpable purpura, Hemorrhagic purpura, Elbow and ankle arthritis, Abdominal pain, Hematuria, Gastrointestinal bleeding

Case 4	6 yr	Male	Palpable purpura, Gastrointestinal bleeding, Abdominal pain, Arthralgia

Case 5	7 yr	Female	Proteinuria, Hematuria, Palpable purpura, Hemorrhagic purpura, Arthritis, Abdominal pain, Gastrointestinal bleeding, Generalized edema

Case 6	18 month	Male	Diffuse purpuric lesions, Gastrointestinal bleeding, Severe abdominal pain, Microscopic hematuria

**Table 2:** Laboratory data and ultrasonographic findings of patients. *Only qualitative measurement of CRP was available at our center.

	WBC/ mm^3^	Hemoglobin gr/dL	Platelet/ mm^3^	CRP*	ESR1st hr	PT/PTT	ANCA	ASO (normal <125 U) Todd unit	BUN mg/dL	Creatinine mg/dL	Urinalysis	Abdominal ultrasonography
Case 1	8500	12.1	253000	++	55	Normal	Neg.	250	12	0.6	Normal	Normal
Case 2	7600	11.6	374000	+	61	Normal	Neg.	166	10	0.5	Many RBC	Normal
Case 3	10500	11.8	320000	+++	42	Normal	Neg.	Neg.	11	0.7	Many RBC	Normal
Case 4	9400	12.2	288000	+	29	Normal	Neg.	333	14	0.5	Normal	Normal
Case 5	11700	12.4	425000	+++	74	Normal	Neg.	Neg.	12	0.6	Protein: ++++ Many RBC	Normal
Case 6	7800	11.6	301000	Neg.	18	Normal	Neg.	Neg.	13	0.5	Normal	Normal

**Table 3:** Treatment protocols and outcomes of patients.

	Corticostroid Dose/Route/Duration	Response to corticostroid	Mycophenolate mofetil (MMF) Dose/Duration	Response to Mycophenolate mofetil (MMF)	Mean arterial pressure (mmHg) before treatment by Mycophenolate mofetil (MMF)	Mean arterial pressure (mmHg) one week after treatment by Mycophenolate mofetil (MMF)
Case 1	Prednisolone 2 mg/kg/d PO for 8 weeks	Stroid dependency	30 mg/kg For 3 weeks	Significant recovery in 48 hours	84	86
Case 2	Prednisolone 2 mg/kg/d PO for 8 weeks	Stroid dependency	30 mg/kg For 6 weeks	Significant recovery in one week	78	77
Case 3	Prednisolone 2 mg/kg/d PO for 8 weeks	Stroid dependency	30 mg/kg For 8 weeks	Significant recovery in one month	80	80
Case 4	Prednisolone 2 mg/kg/d PO for 2 weeks	No response	30 mg/kg For 8 weeks	Significant recovery in one month	86	85
Case 5	Methyl prednisolone 20 mg/kg IV for 3 doses every other day then Prednisolone 2 mg/kg/d for first 4 weeks and 2 mg/kg/every other day for second 4weeks	No response	30 mg/kg For 6 month	Significant recovery of proteinuria but continuation of microscopic hematuria	86	90
Case 6	Prednisolone 2 mg/kg/d PO for first 4 weeks and 2 mg/kg/every other day for second 4weeks	Stroid dependency	30 mg/kg For 8 weeks	Significant recovery in one month	76	79
